# A Review of Ribonuclease 7’s Structure, Regulation, and Contributions to Host Defense

**DOI:** 10.3390/ijms17030423

**Published:** 2016-03-22

**Authors:** Brian Becknell, John David Spencer

**Affiliations:** 1 Center for Clinical and Translational Research, The Research Institute at Nationwide Children’s, Columbus, OH 43205, USA; brian.becknell2@nationwidechildrens.org; 2 Division of Pediatric Nephrology, Nationwide Children’s, Columbus, OH 43205, USA

**Keywords:** ribonuclease, antimicrobial peptides, ribonuclease 7, innate immunity, infection, host defense

## Abstract

The Ribonuclease A Superfamily is composed of a group of structurally similar peptides that are secreted by immune cells and epithelial tissues. Several members of the Ribonuclease A Superfamily demonstrate antimicrobial activity, and it has been suggested that some of these ribonucleases play an essential role in host defense. Ribonuclease 7 (RNase 7) is an epithelial-derived secreted peptide with potent broad-spectrum antimicrobial activity. This review summarizes the published literature on RNase 7’s antimicrobial properties, structure, regulation, and contributions to host defense. In doing so, we conclude by highlighting key knowledge gaps that must be investigated to completely understand the potential of developing RNase 7 as a novel therapeutic for human infectious diseases.

## 1. Introduction

Antimicrobial peptides (AMPs) are one of the most primitive components of the immune system and are arguably the most widely used type of host defense molecule in nature. Although these peptides were originally identified in lower phyla like plants, insects, and amphibians, AMPs play important roles in the mammalian immune response. To date, over 2,650 AMPs have been described from six kingdoms—including bacteriocins from bacteria, archaea, protists, fungi, plants, and animals. In humans, 112 host defense peptides have been identified [1]. Despite their ancient lineage, AMPs have remained effective as antimicrobials, confounding the perception that pathogens develop resistance to every conceivable antimicrobial substance. AMPs are conventionally defined as polypeptide antimicrobials, encoded by genes and synthesized by ribosomes. This definition distinguishes AMPs from traditional antibiotics like penicillin, which is a product of secondary metabolism from bacteria and fungi [2,3].

AMPs predominantly kill pathogens by disrupting their membranes. AMPs rapidly kill both Gram-positive and Gram-negative pathogens. Their antimicrobial activity is dependent on their positive charge, secondary structure, disulfide bonding, and amphipathicity. The net positive charge, as indicated by a high isoelectric point (pI), promotes binding to negatively charged microbial phospholipid membranes. Amphipathicity permits AMPs to achieve high concentrations in aqueous environments or within a membrane, a key requisite to disrupt microbial lipid bilayers. Secondary structure motifs, including α-helices and β-sheets, promote pore formation in microbial membranes while disulfide bridges maintain the peptide’s structural integrity [4,5]. AMPs retain their antimicrobial activity as long as their cationic charge, amphipathicity, and secondary structure are preserved. Tissues that routinely encounter microbes, including epithelial cells and white blood cells, are the primary AMP producers. In humans and mammals, cathelicidins, defensins, and ribonucleases are the major AMP families [6].

The mammalian Ribonuclease A Superfamily consists of several peptides found in a range of mammals. RNase A, initially isolated from bovine pancreas, is the best-characterized ribonuclease. It has served as a model peptide in work related to protein folding, disulfide bond formation, protein crystallography and spectroscopy, and protein dynamics. Classic experiments on the denaturation and renaturation of this protein appear in almost every biochemistry textbook [7]. Since the period of its discovery, methods for detecting ribonuclease activity improved, which lead to the identification of several ribonucleases in the RNase A Superfamily. As a group, RNase A Superfamily members are secretory peptides that include a classic hydrophobic signal peptide followed by a mature peptide. Mature peptides in this family are ~12–16 kDa in size and contain 6–8 cysteine residues that form 3–4 disulfide bonds which play an important role in maintaining their tertiary structure [8,9].

In humans, the RNase A Superfamily was originally thought to contain eight peptides on chromosome 14q11.2. These canonical ribonucleases, which contain the catalytic motif (CKXXNTF), include pancreatic RNase (also known as RNase 1), eosinophil-derived neurotoxin (EDN/RNase 2), eosinophil cationic protein (ECP/RNase 3), RNase 4, angiogenin (RNase 5), RNase 6 (k6), RNase 7, and RNase 8 [10]. However, analysis of the human genome identified five additional noncanonical ribonucleases, which are named RNase 9–13 [11,12]. The structure and enzymatic activity of many RNase A Superfamily members have been well characterized. Besides possessing ribonucleolytic activity, ribonucleases in this family have been shown to play roles in angiogenesis, neurotoxicity, immunomodulation, and host defense [4,9,10,13,14]. On a per molar basis, RNase 7 has been identified as one of the most potent human antibacterial ribonucleases, making it an ideal candidate to study as a novel therapeutic against antibiotic resistant pathogens [4]. Thus, this review focuses on the antimicrobial properties of RNase 7 and its contributions to host defense.

## 2. Discovery and Characterization of the RNase 7 Peptide

When analyzing healthy human skin for the presence of endogenous AMPs, Harder and Schroeder identified a novel 14.5 kDa peptide which they called RNase 7. Harder and Schroeder demonstrated that natural skin-derived RNase 7 exhibits potent *in vitro* antimicrobial activity against Gram-positive bacteria, Gram-negative bacteria, and the yeast *Candida albicans* [15]. Around the same time, Zhang and colleagues [7] identified the corresponding *RNASE7* gene when performing a computational search of the human genome database. They expressed recombinant RNase 7 in *Escherichia coli* (*E. coli*) and demonstrated that it inhibited bacterial growth in the low micromolar concentration range. Recombinant RNase 7 did not demonstrate antiviral activity.

Using standardized assays that evaluate yeast tRNA degradation, both research groups demonstrated that RNase 7 has high ribonuclease activity (about 50-fold greater than ECP/RNase 3) [7,15]. However, RNase 7’s ribonuclease activity may not be required for its antimicrobial activity. Huang *et al* [16] generated ribonuclease-inactive recombinant RNase 7 peptides by mutating catalytic histidines and lysines (H15A, K38A, H123A) and demonstrated comparable antimicrobial activity to wild-type RNase 7 against *Pseudomonas aeruginosa (P. aeruginosa)*. Similarly, Harder and colleagues found that a ribonuclease-deficient RNase 7 mutant (H123A) retained antimicrobial activity against *Enterococcus faecium* (*E. faecium*) and *E. coli*—confirming that the antibacterial effects of RNase 7 function independently of its ribonuclease activity against these pathogens [17].

**Figure 1:**
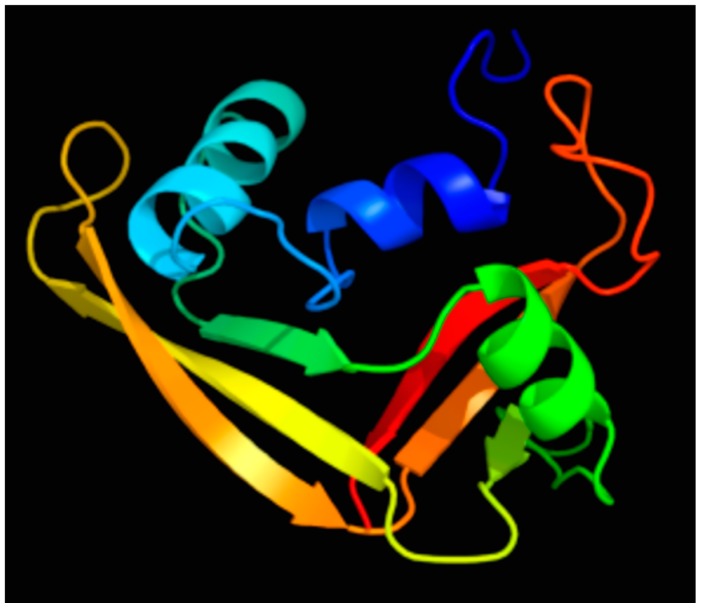
Predicted solution structure of RNase 7 colored by rainbow spectrum. The N-terminus is depicted by the blue color and the C-terminus is depicted by the red color. Figure adapted from [20,21].

When evaluating the solution structure of RNase 7 (Figure 1), Huang and colleagues identified three clusters of cationic residues on RNase 7’s surface (composed of lysine and arginine). They demonstrated that a cluster of lysine residues at the flexible coil near RNase 7’s N-terminus—K(1), K(3), K(111), K(112)—is critical for bactericidal activity [16]. Recently, Wang and colleagues [18] implicated the N-terminus of RNase 7 as a key domain for microbicidal activity toward uropathogenic *E. coli*, *Staphylococcus saprophyticus* (S. *saprophyticus*), and *Proteus mirabilis*. An N-terminal fragment of RNase 7 (residues 1–97) displayed up to 4-fold greater potency against *E. coli* and *S. saprophyticus* compared to full-length RNase 7 peptide. Similarly, using chemically synthesized peptides, Torrent *et al* [19] demonstrated that the antimicrobial activity for human canonical RNases with antimicrobial function is retained at the N-terminus and that the mechanism of action of the N-terminal domains is similar to that of the full-length proteins. Moreover, using computational analysis, they showed that the antimicrobial propensity for all vertebrate RNases is conserved at the N-terminus, thereby suggesting that the N-terminal domain may have been evolutionarily selected to provide a host–defense function.

## 3. RNase 7’s Bactericidal Mechanisms

How does RNase 7 kill microbes? Using atomic force microscopy, we visualized bacterial membrane splitting and bleb formation on the surfaces of *E. coli*, *P. aeruginosa*, and *E. faecalis* after treatment with recombinant RNase 7, confirming its ability to disrupt bacterial structural integrity [22]. Using biophysical and microscopy methodologies, Torrent and colleagues investigated RNase 7’s membrane destabilizing capabilities. They simulated RNase 7’s interaction with microbial plasma membranes using phospholipid vesicles. RNase 7’s antimicrobial mechanisms were compared to recombinant RNase 3, the most studied human antimicrobial ribonuclease [23]. Their results demonstrate that the mechanisms of RNase 3 and RNase 7 are electrostatically driven. However, each peptide uses distinct mechanisms to disrupt lipid bilayers. While RNase 3 triggers vesicle aggregation, RNase 7 induces local membrane destabilization well before aggregation occurs [24]. In subsequent studies, this same research group evaluated the effects of RNase 3 and RNase 7 on the microbial cell wall. Both RNase 7 and RNase 3 display high affinity for Lipopolysaccharide (LPS) and peptidoglycan (PGN) at the Gram-negative and Gram-positive outer surfaces. Prior to causing cell lysis and death, RNase 3 aggregates *E. coli* and *S. aureus*. In contrast, RNase 7 does not share this activity and elicits release of bacterial cell contents without causing bacterial aggregation [25]. Together, these results suggest that RNase 7’s antimicrobial activity is dependent on its ability to disrupt the bacterial cell rather than interacting with internal microbial targets. In part, these findings support the hypothesis that ribonuclease activity is not required for RNase 7’s antimicrobial action. These results also indicate that further studies are warranted to identify the microbial surface proteins targeted by RNase 7. Recent studies demonstrate that RNase 7 complexes with outer membrane protein I (OprI) on the surface of *P. aeruginosa* and His-tagged outer membrane Lipoprotein (Lpp), a major surface protein of *E.coli* and *Enterobacteriaceae* [26,27].

## 4. RNase 7 Expression and Roles in Host Defense

As noted, RNase 7 was originally isolated from stratum corneum skin extracts.

**Figure 2:**
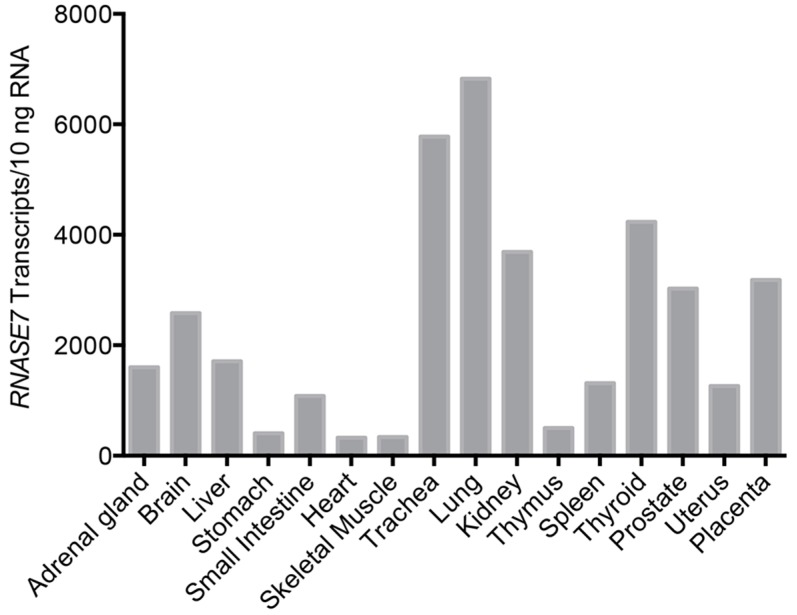
Tissue distribution of *RNASE7* mRNA expression. RNA from various human tissues was reverse transcribed and *RNASE7* gene expression was analyzed by quantitative real-time PCR. Figure adapted from [21].

**Figure 3:**
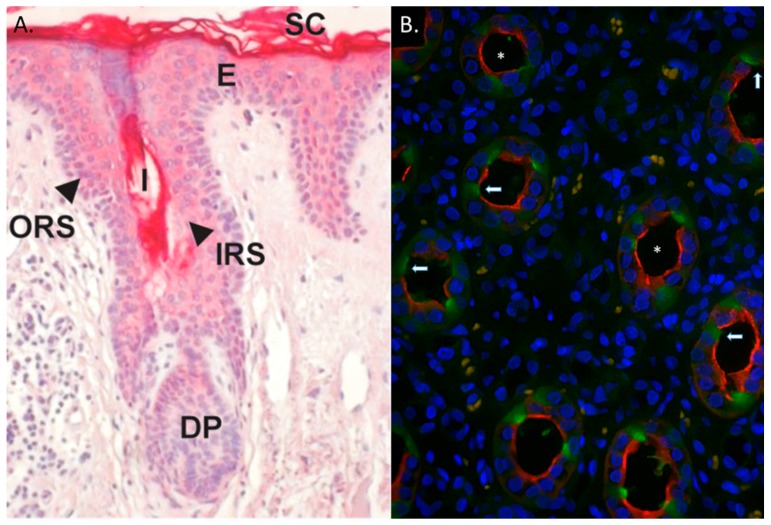
RNase 7 expression in human skin and kidney. (**A**) Immunostaining of RNase 7 peptide in human skin demonstrates strong RNase 7 expression in the upper epidermal layers. Hair follicles also stained positively. SC: Stratum corneum, E: Epidermis; ORS: Outer Root Sheath; IRS: Inner Root Sheath; I: Infundibulum; DP: Dermal Papilla. Magnification 20×. Panel A was adapted from [17]; (**B**) Immunoflourescence of human kidney labeled for RNase 7 (green/arrows), nuclei (blue), and aquaporin-2 (AQP-2). AQP-2 (red) labels principal cells in the collecting tubule. Principal cells (red) were negative for RNase 7 (green), indicating that the intercalated cells of the collecting tubules produce RNase 7. The asterisk (*) identifies the urinary space. Magnification 40×.

However, additional tissues also express RNase 7—with the most abundant mRNA expression in respiratory and urinary tracts (Figure 2) [7,15,28]. Recent evidence also suggests that RNase 7 is also one of the main AMPs expressed in articular joints, the oral cavity, the cornea, and basal respiratory epithelial cells [29,30,31,32]. Northern analysis did not detect *RNASE7* mRNA in blood leukocytes [7]. In addition, our research group has not detected *RNASE7* transcripts in human monocytes, neutrophils, or NK cells (unpublished observation). In the skin, *RNASE7* is the most highly expressed RNase A Superfamily member. *RNASE7* mRNA expression is greater than other AMPs—including human defensin 2, psoriasin (S100A7), and cathelicidin. The outermost, more differentiated epidermal layers produce RNase 7 peptide, indicating that RNase 7 production is greatest where microbial insult most likely occurs [17,33]. Similarly, in hair follicles, RNase 7 expression is greatest in the outer root sheath suggesting a role for RNase 7 in protecting the hair follicle from microbial challenge (Figure 3A) [17,34].

Our research group has identified a similar RNase 7 expression pattern in the urinary tract. RNase 7 is produced by the bladder urothelium and secreted into the urine at high concentrations [22,28]. In the kidney, the intercalated cells of the collecting tubules produce RNase 7 (Figure 3B). Given the physiological position of the collecting tubule, intercalated cells are ideally positioned to defend the kidney from an ascending urinary tract infection (UTI), as they are the initial cell types specifically targeted by ascending microbes before they infiltrate the renal parenchyma [28,35].

In the skin and urinary tract, the biological relevance of RNase 7 has been evaluated using antibody neutralization assays. Incubation of stratum corneum extracts with an RNase 7-neutralizing antibody reduced the killing activity toward *E. faecium* [17]. Similarly, application of an RNase 7-neutralizing antibody to the surface of skin explants enhanced the growth of *S. aureus* [36]. The antimicrobial activity of urine was also suppressed by the addition of an RNase 7-neutralizing antibody [28,37]. Overall, these data implicate RNase 7 in maintaining epithelial sterility, consistent with the proposed role of RNase 7 in innate immunity and antimicrobial defense.

## 5. RNase 7 Induction and Regulation

Although RNase 7 is constitutively expressed at high concentrations, its expression can be induced. Compared to control samples, RNase 7 peptide concentrations have been shown to be higher in patients with psoriatic skin lesions, atopic dermatitis, dermatophyte skin infections, and urinary tract infections [22,38,39,40,41]. *In vitro* studies demonstrate that the treatment of primary keratinocytes with interleukin (IL)-17A, tumor necrosis factor-α, IL-1β, interferon-γ, a dermatophyte (*Trichophyton rubrum*), or bacteria induces RNase 7 expression [15,30,36,42,43,44,45]. Bacterial biofilms have also been shown to induce RNase 7 in gingival epithelial cells, and protozoa induced RNase 7 in corneal epithelial cells [46,47].

To date, the molecular mechanisms in which these heterogeneous stimuli induce RNase 7 expression are not well characterized. Using immunohistochemistry, Reithmayer *et al* [34] demonstrate that that RNase 7 is produced in human hair follicles in response to lipoteichoic acid (LTA), protein A, or LPS—suggesting the involvement of Toll-Like Receptor (TLR)-mediated pathways in RNase 7 regulation. Wanke and colleagues have shown that skin commensal and pathogenic bacteria differ in their ability to induce RNase 7 expression in primary human keratinocytes. While commensal *Staphylococcus* strains (*S. epidermidis*) induce RNase 7 expression via TLR-2, epidermal growth factor receptor (EGFR), and nuclear factor-κB activation, pathogenic *Staphylococci* (*S. aureus*) activate mitogen-activated protein kinase (MAPK) and phosphatidylinositol 3-kinase/AKT signaling pathways and suppress NF-κB activation. Similarly, Mohammed *et al.* [30] have shown that MAPK signaling, independent of NF-κB, modulates RNase 7 expression in response to IL-1β stimulation in SV40-transformed human corneal epithelial cells. Firat and colleagues demonstrated that *Trichophyton rubrum*, induces RNase 7 expression via EGFR [45]. Finally, Simanski *et al* found that IL-17A and interferon gamma synergistically induce *RNASE7* mRNA expression via Signal Transducer and Activator of Transcription (STAT)-3 in primary human keratinocytes [42].

Several recent studies add to the complexity of RNase 7 regulation. (1) Mun and colleagues found that tear fluid induces microRNA (miR)-762 expression in corneal epithelial cells, which negatively regulates RNase 7 levels. It is not known if miR-762 directly regulates RNase 7 expression, but the authors of this study argued this was unlikely based on comparison of the miR-762 seed region and *RNASE7* mRNA [31]. (2) Amatngalim *et al.* [32] found that cigarette smoke induces epithelial injury in primary bronchial epithelial cells, leading to EGFR-dependent induction of RNase 7 expression by the basal layer. The authors of this study suggest that RNase 7 may provide a second line of defense in basal cells, following airway epithelial injury. Together, these findings suggest that RNase 7 is expressed in response to a range of stimuli in tissues that play a role in host defense. However, the precise physiological role that RNase 7 plays and the signaling mechanisms involved still unclear.

## 6. Regulation of RNase 7 by the Ribonuclease Inhibitor

Recent studies have suggested that the ribonuclease inhibitor (RI) regulates RNase 7 activity. RI is an abundant, 50 kDa cytosolic protein found in all mammals. Human RI contains 15 leucine-rich repeats that form a horseshoe-like structure capable of reversibly binding to several members of the RNase A superfamily members in a 1:1 molar ratio with high affinity [48].

Abtin and colleagues [33] demonstrated that RI blocks recombinant RNase 7 antimicrobial and ribonuclease activities *in vitro*. Whereas RNase 7 is abundant in the most superficial layers of skin (stratum corneum), RI is absent. In addition, protein extracts from stratum corneum exhibits protease activity towards RI. Given these findings, these investigators concluded that skin proteases degrade RI to liberate RNase 7 to shield the epidermis from microbial challenge.

Our research group also investigated the relationship between RNase 7 and RI in the urinary tract [37]. Similar to Abtin *et al* [33], we found that RI inhibits the antimicrobial activity of RNase 7 toward bacterial uropathogens. Additionally, we demonstrated that RI binding to RNase 7 reduces RNase 7’s affinity for bacterial cell wall components. Like RNase 7, RI is highly expressed in the bladder, where both proteins localize to superficial urothelial cells. Within the kidney, RI is expressed along the basolateral surfaces of the intercalated cells, while RNase 7 localizes to the apical surfaces of these same cell types. RNase 7 and RI interact directly as recombinant proteins and *in vivo* by immunoprecipitation-Western blotting. While kidneys from individuals with pyelonephritis have increased RNase 7 mRNA expression, RI mRNA expression is reduced compared to uninfected controls [37]. This may occur in response to oxidative stress or proteolysis resulting from leukocyte recruitment. These findings raise the question of whether the RNase 7–RI interaction is tightly regulated *in vivo*.

## 7. Prospective

Over the past decade, considerable progress has been made defining the expression and antimicrobial activity of RNase 7. However, several key questions remain unsolved regarding RNase 7’s antimicrobial activity, regulation, and function *in vivo*. The answers to some of these questions will pave the way to understanding the potential of RNase 7 as a novel therapeutic.

### 7.1. Does RNase 7 Play a Significant Role in Host Defense?

Currently, there is indirect evidence that evaluates RNase 7’s contribution to host defense [17,28]. Experimental studies that evaluate RNase 7’s *in vivo* function are limited, in part, by its restricted expression to primates. Thus, novel models and experimental approaches are needed to truly appreciate the contribution of RNase 7 to host defense *in vivo*.

### 7.2. Does Altered RNase 7 Production Impact Infection Susceptibility?

As we continue to evaluate RNase 7’s contributions to host defense, we will begin to understand if dysregulation of RNase 7 production affects infection susceptibility. To date, it is unknown whether deficient RNase 7 production increases infection risk or if increased RNase 7 production shields the host from microbial challenge. If individuals with recurrent infections or increased infection risk have suppressed RNase 7 activity, it is possible that this could occur secondary to genetic variation, deficient protein production/function, or the ability of pathogens to directly suppress RNase 7 production. In addition, it is not known at what stage of human development RNase 7 production begins and how RNase 7’s antimicrobial barriers mature or weaken with age [49].

### 7.3. What Is the Significance of RNase 7’s Catalytic Activity?

As noted above, the significance of RNase 7’s ribonuclease activity has been called into question by the observation that its bactericidal activity is maintained in catalytically inactive mutants [16,17]. However, it is possible that ribonuclease activity is required for killing of selective pathogens, given the wide range of microbes susceptible to RNase 7. Within the context of the entire Ribonuclease A Superfamily, distinct family members may combine their diverse abilities, including their catalytic activity, to provide a fast and multifaceted mechanism to simultaneously combat pathogens at different cellular targets [19]. Alternatively, the catalytic activity of RNase 7 may have direct effects on the host immune response. Excessive exposure to microbial RNA activates pattern recognition receptors such as TLR-7 and TLR-8 in dendritic cells and monocytes, resulting in type I interferon production, cytokine production, and inflammasome activation [50]. Thus, RNase 7’s catalytic activity may help process microbial RNA and impair their ability to serve as pathogen associated molecular patterns and engage TLRs, thereby attenuating the host immune response. Comprehending the interactions of RNase 7 with host and microbial RNA molecules is therefore of great potential relevance to framing the context for RNase 7’s contributions to host immunity.

### 7.4. What Mechanisms Are essential for RNase 7’s Antimicrobial Properties?

Understanding RNase 7’s bactericidal mechanisms is essential for its development as a novel therapeutic. Because RNase 7 has the ability to disrupt the microbial membrane, it functions differently than conventional antibiotics, which inhibit cell wall synthesis, DNA replication, RNA transcription, or protein synthesis. However, it still remains unclear how RNase 7 specifically targets the microbial cell wall and how it permeabilizes the membrane. Current studies have not investigated whether RNase 7’s interactions with microbial membranes directly cause bacterial lysis or if it triggers a bacterial autolysis process [4]. In addition, it is still unknown whether RNase 7 may contribute to host defense in roles independent of its direct bactericidal properties—degrading extracellular RNA, acting as a chemokine, or serving as an opsonin by binding extracellular nucleic acids and promoting interactions with other innate immune receptors. Recent reviews on AMPs indicate that they have multifunctional effects [9,51].

### 7.5. What Molecular Processes Regulate RNase 7 Expression?

As noted above, there is limited published evidence investigating the mechanisms that regulate RNase 7 expression. Future studies are needed to elucidate the molecular mechanisms, signal transduction pathways, and transcription factors that control basal RNase 7 expression and RNase 7 expression during microbial challenge. Moreover, consideration should also be given to assessing how RI regulates RNase 7 activity. Currently, it is unclear if RI is proteolytically inactivated in the setting of bacterial infection, nor is it apparent how the interactions of RNase7 and RI are modulated in this setting.

Alternatively, additional investigations are needed to identify if RNase 7 undergoes post-translational modifications including acetylation, glycosylation, and phosphorylation. For example, RNase 3/ECP undergoes variable degrees of *N*-glycosylation, and heavily glycosylated ECP possesses reduced antimicrobial activity [52]. The published literature has not yet accounted for the presence and significance of these covalent modifications. Whether R7/RI are regulated at the post-translational level *in vivo* requires investigation.

### 7.6. Will We Realize the Therapeutic Potential for RNase 7?

AMPs have great therapeutic potential for treatment of human infectious disease, though this vision has not yet been realized. Although RNase 7 exhibits potent antibacterial properties as an AMP *in vitro*, additional studies are required to confirm these observations *in vivo* and to assure that increased levels or activity of RNase 7 are not toxic to the host. Such studies may reveal additional roles for RNase 7 as a cytoprotective agent in the setting of mucosal injury. While chemical synthesis of RNase 7 derived peptides and production of recombinant RNase 7 protein is formally possible, the most practical and safest approach to RNase 7-directed therapies may lie in induction of endogenous RNase 7 by as-yet-unidentified natural products or approved pharmaceuticals. Additional strategies to increase RNase 7 activity may include: (1) use of RNase 7 stabilizing agents that prolong its longevity *in vivo*; and (2) methods to destabilize the RNase7/RI complex, thereby enhancing RNase 7 AMP activity. Clearly, the path to therapeutic application for RNase 7 is not well defined at this point, and demands better clarification of RNase 7’s underlying function and regulation *in vivo*.
